# COPD Management in Primary Care: Underutilisation of Nursing Consultations

**DOI:** 10.1111/jocn.70241

**Published:** 2026-02-12

**Authors:** Marc Vila, Meritxell Mondejar, Sergio Cazorla‐Calderón, Àngels Ballarin, Sandra Casas‐Recasens, Rosa Faner, Alvar Agustí

**Affiliations:** ^1^ Equip d'Assistència Primària Vic (EAPVIC) Barcelona Spain; ^2^ Faculty of Health Sciences and Welfare, Research Group M3O, Methodology, Methods, Models and Outcomes of Health and Social Sciences University of Vic‐Central University of Catalonia Barcelona Spain; ^3^ Catedra Salud Respiratoria University of Barcelona Barcelona Spain; ^4^ Institut d'Investigacions Biomèdiques August Pi i Sunyer (IDIBAPS) Barcelona Spain; ^5^ CIBER Enfermedades Respiratorias (CIBERES) Instituto de Salud Carlos III (ISCIII) Madrid Spain; ^6^ Department of Immunology University of Barcelona Barcelona Spain; ^7^ Department of Pulmonology Respiratory Institute, Hospital Clínic Barcelona Barcelona Spain

**Keywords:** care plan, COPD, healthcare utilisation, nursing, primary care

## Abstract

**Objective:**

To describe the clinical profile, comorbidity burden, follow‐up and healthcare utilisation in patients labelled as having Chronic Obstructive Pulmonary Disease (COPD) in Primary Care (PC) nursing consultations.

**Design:**

Real‐world data COPD, retrospective, observational study using routinely collected data in electronic health records (EHR). This study adheres to the STROBE reporting guidelines for cross‐sectional studies.

**Location:**

Three Primary Care centres in Catalonia, Spain, belong to the Catalan Health Service.

**Participants:**

All patients aged ≥ 15 years with a recorded diagnosis of COPD in their EHR, excluding institutionalised individuals and those deceased before study onset. Final sample: 474 patients (105 women, 369 men; mean age 70 years) from a reference population of 28,000 individuals.

**Main Measurements:**

Data included socio‐demographics, smoking/alcohol, mMRC dyspnea, inhaled therapy/adherence, spirometry, comorbidities, Adjusted Morbidity Groups (GMA), active COPD care plans and 12‐month healthcare use.

**Results:**

EHR showed a high rate of missing data in follow‐up variables (inhaler adherence 28.5%; dyspnea 17%–20%). Despite that, all participants were ‘labelled’ as COPD, most of them lacked spirometric confirmation. Active smoking was highly prevalent (52.3% women, 45.0% men). Hypertension, obesity and osteoarthritis were the most common comorbidities; anxiety, depression, osteoporosis and thyroid disorders were more frequent in women. Higher GMA complexity correlated with more Primary Care visits, especially nursing consultations, particularly in patients with cardiovascular disease and diabetes (*p* < 0.001) for 12 months follow‐up. No significant differences between groups were found in urgent or hospital care use.

**Conclusions:**

EHR‐labelled COPD patients with cardiometabolic comorbidity received more structured nursing follow‐up and more annual visits than without. Improving EHR recording, integrating spirometry with the EHR, and prioritising high‐complexity profiles could enhance monitoring, treatment optimisation and equity—nursing consultations are a key lever.

**Patient or Public Contribution:**

No patients or members of the public were directly engaged in the study design or data analysis. Nevertheless, the research was motivated by patient needs and aims to improve healthcare services.

## Key Points

**What Is Already Known About This Topic?:**

- Many patients in primary care are labelled as having COPD without spirometric confirmation.
- COPD is frequently associated with multimorbidity and high healthcare use, particularly in older adults.

**What This Paper Adds?:**

- Most EHR‐labelled COPD patients lacked spirometric confirmation and showed relevant gaps in nursing follow‐up records.
- Cardiometabolic comorbidity and higher GMA complexity were associated with more frequent primary care, especially nursing, consultations.
- Important sex differences were observed in comorbidity patterns amongst patients labelled as having COPD.

**Implications for Practice:**

- Improving EHR data quality and integrating spirometry into routine records is essential for accurate COPD management.
- Nursing consultations play a key role in monitoring complex COPD profiles and should be prioritised in patients with higher morbidity burden.

## 1 Introduction

Chronic obstructive pulmonary disease (COPD) is a major public health problem due to its high prevalence (10% in the population over 40 years of age) (Soriano et al. 2021; Fletcher and Peto 1978), increasing incidence (related to population ageing), associated morbidity and mortality (it is currently the third leading cause of death worldwide) (GOLD 2025a) and high social and economic impact (Lange et al. 2015). COPD is a preventable and treatable disease, but there is a high rate of underdiagnosis (70%–80% of COPD patients worldwide remain undiagnosed (Soriano et al. 2021)) and misdiagnosis (30%–60% of patients labelled as having COPD do not show persistent airflow obstruction on forced spirometry, which is the required diagnostic criterion (Diab et al. 2018)). Both are significant clinical problems because underdiagnosis implies absence of treatment, while misdiagnosis leads to inappropriate treatment (Diab et al. 2018; Aaron et al. 2024; Vila et al. 2025).

Nursing consultations in Primary Care (PC) can provide an opportunity to improve the management and follow‐up of COPD patients, to identify those who are undiagnosed or misdiagnosed, to assess the treatment received and the correct use of inhalers, as well as to initiate preventive therapeutic interventions (e.g., smoking cessation). The Preventive Activities and Health Promotion Programme (PAPPS) of the Spanish Society of Family and Community Medicine (SEMFyC) provides a structured framework for these interventions (Guillem 2022; Córdoba García et al. 2022).

However, there is limited information on the activity of nursing consultations in primary care within the framework of the PAPPS programme. This gap particularly affects patients recorded as having COPD in the Electronic Health Record (EHR), without systematic spirometric confirmation. As a result, the clinical characteristics of these patients remain poorly described. Therefore, in this study we sought to evaluate the clinical profile and level of complexity, type of documentation available in their EHR, and use of healthcare resources (visits, medications) amongst patients recorded as having COPD in primary care in Catalonia, Spain.

## 2 Methods

### 2.1 Study Design and Setting

This retrospective observational study used real‐world data extracted from the electronic health records (EHRs) of patients recorded as having COPD in three primary care centres (ABS–EAP Vic, El Remei, Santa Eulàlia de Riuprimer and Muntanyola), located within a Basic Health Area in a semi‐urban region of Osona, Central Catalonia, Spain. In the Spanish healthcare system, all nationals are assigned to a PC centre, where they can receive comprehensive management of the most common diseases by General Practitioners (GP) and nurses. The EHR collects a wide range of clinical information—such as diagnoses, health problems and clinical indicators—recorded by these health care professionals (HCP) during routine clinical visits. This retrospective study used routinely collected EHR data and was reported in accordance with the STROBE guidelines for observational studies.

### 2.2 Study Population

The study population included 28,000 individuals aged 15 years or older assigned to these three PC centres. The age threshold (≥ 15 years) was chosen because it corresponds to the transition from paediatric to adult care in the Spanish healthcare system, where COPD‐related follow‐up and diagnostic coding are managed in adult primary care (Willis 2020). All patients labelled as COPD, this is with a recorded diagnosis of COPD according to the International Classification of Diseases, 10th Revision (ICD‐10), codes J43.0–2, J43.8–9, J44.0–1 and J44.8–9, were included in the analysis. Institutionalised individuals (e.g., nursing home residents) and patients with COPD who had died before the study start date were excluded.

### 2.3 Data Extracted and Variables Analysed

Data from the EHRs of patients with a recorded diagnosis of COPD were extracted through a structured language (SQL) search, retrieving the following variables if available (Vila et al. 2025):

- Sociodemographic variables: age, sex, weight, height and body mass index (BMI).
- Smoking (current smoker, cigarettes/day, pack‐years) and alcohol consumption (yes/no)
- Degree of dyspnea assessed using the modified Medical Research Council (mMRC) scale, using the most recent value recorded in the EHR.
- Active pharmacological treatment prescribed for COPD and inhaler adherence recorded in the EHR, based on clinical assessment by healthcare professionals during routine visits in the 12 months prior to the index date.
- Spirometric data (FEV_1_, FVC and FEV_1_/FVC ratio, both pre‐ and post‐bronchodilation) most recently stored in the designated EHR fields, excluding free text entries. Their absence does not confirm that spirometry was not performed, but rather that it was not recorded in the structured EHR fields.
- Most frequent comorbidities (cardiovascular, gastrointestinal, endocrinological, musculoskeletal, neurological, respiratory [other than COPD], anxiety/depression and renal failure) and total number of chronic diseases, identified using ICD‐10 diagnostic codes extracted from structured fields of the EHR.
- Adjusted Morbidity Groups (AMG) index, which provides a weighted summary of chronic disease morbidity, acute diagnosis codes and complexity (Monterde et al. 2016).
- Active COPD care plans (Meehan et al. 2020; Kara 2007), defined as documented and ongoing COPD‐specific care plans in the EHR, reflecting active nursing follow‐up and including secondary prevention activities (e.g., smoking cessation counselling, inhaler technique assessment and vaccination advice). Physical activity was recorded in the EHR using a non‐standardised field, without specification of intensity, frequency or duration. As no validated instrument or predefined criteria were available, this variable was considered non‐interpretable and was therefore reclassified as missing for analytical purposes.
- Healthcare utilisation data from the previous year, including visits to PC physicians and/or nurses and hospital services (home ambulance calls, emergency department visits and hospital admissions).

### 2.4 Statistical Analysis

All statistical analyses were performed using R (version 4.2) (R Core Team 2024) with customised scripts. Given the retrospective and descriptive nature of the study and the use of real‐world data extracted from electronic health records (EHRs), analyses were conducted using available‐case analysis only. No data imputation was performed.

Categorical variables are presented as numbers and percentages, and continuous variables as mean ± standard deviation. Comparisons between groups were performed using non‐parametric tests, including the Kruskal–Wallis test for continuous variables and the Chi‐square or Fisher's exact test for categorical variables, as appropriate. Non‐parametric methods were selected due to the absence of distributional assumptions and the presence of missing data.

No adjustment for potential confounders was undertaken, as the study was not designed to assess causal associations. Likewise, no correction for multiple comparisons was applied, given the exploratory and descriptive aim of the analyses. Effect sizes and confidence intervals were not calculated, as the study did not aim to estimate effects but to describe patterns of clinical characteristics and healthcare utilisation.

## 3 Results

### 3.1 General Characteristics of the Study Population

A total of 474 patients labelled as having COPD in the EHR (105 women and 369 men) were included in the analysis, with a similar mean age (69 and 70 years, respectively) (Table 1). Given that the population assigned to the three analysed PC centres is 28,000 individuals, the proportion of ‘patients labelled as COPD’ in this database was only 1.7%. It should also be noted that many data fields were incomplete in the EHR; therefore, the values shown in Table 1 correspond only to those recorded in the database (as shown). These two observations highlight that the first issue identified in this analysis is the lack of complete recording of relevant variables in the EHR.

**TABLE 1:** Clinical characteristics of the study population by sex.

	All	Women	Man	*p*
	*N* = 474	*N* = 105	*N* = 369	Women vs. man
Demographics				
Age	69.8 (11.2)	69.1 (10.2)	70.0 (11.5)	0.465
Weight, (*n* = 469)	77.5 (16.0)	67.4 (13.5)	80.5 (15.5)	< 0.001**
Height, (*n* = 472)	168 (9.16)	158 (6.67)	171 (7.53)	< 0.001**
BMI, (*n* = 471)	27.6 (5.10)	27.3 (5.79)	27.7 (4.89)	0.53
Physical activity, *n* (%)				0.221
Sufficiently active	429 (99.8%)	94 (98.9%)	335 (100%)	
Sedentary	1 (0.23%)	1 (1.05%)	0 (0.00%)	
Tobacco				
Current smoker, *n* (%)	189 (46.6%)	46 (52.3%)	143 (45.0%)	0.274
Pack‐years	30.3 (27.2)	6.58 (9.31)	36.3 (27.0)	< 0.001**
Tobacco intervention, *n* (%)				0.048*
Behavioural reinforcement	110 (31.7%)	18 (25.0%)	92 (33.5%)	
Motivational interview	11 (3.17%)	1 (1.39%)	10 (3.64%)	
Brief intervention	9 (2.59%)	4 (5.56%)	5 (1.82%)	
Intensive intervention	197 (56.8%)	41 (56.9%)	156 (56.7%)	
Group intervention	20 (5.76%)	8 (11.1%)	12 (4.36%)	
Smokin cessation stage, *n* (%)				0.94
Pre‐contemplative	40 (67.8%)	8 (66.7%)	32 (68.1%)	
Contemplative	5 (8.47%)	1 (8.33%)	4 (8.51%)	
Action	7 (11.9%)	1 (8.33%)	6 (12.8%)	
Relapse	7 (11.9%)	2 (16.7%)	5 (10.6%)	
Active treatment (Bupropion/Cytisine Nicotine)	7 (1.5%)	1 (1%)	6 (1.62%)	0.429
Alcohol consumption, *n* (%)				< 0.001**
Abstinent	239 (52.1%)	75 (75.0%)	164 (45.7%)	
Low risk	195 (42.5%)	25 (25.0%)	170 (47.4%)	
Moderate risk	24 (5.23%)	0 (0.00%)	24 (6.69%)	
High risk	1 (0.22%)	0 (0.00%)	1 (0.28%)	
Clinical variables				
Respiratory rate (*n* = 86)	23.4 (15.4)	21.0 (5.18)	23.9 (16.7)	0.23
O2 Saturation, (*n* = 211)	95.7 (2.36)	95.8 (2.25)	95.7 (2.39)	0.703
HR, (*n* = 464)	75.8 (14.6)	78.6 (13.0)	75.0 (14.9)	0.02*
BP (systolic), (*n* = 472)	131 (14.6)	128 (14.5)	131 (14.6)	0.09
BP (diastolic), (*n* = 472)	77.2 (9.40)	77.6 (8.89)	77.1 (9.55)	0.591
Dyspnea scale (mMRC), *n* (%)				0.872
0	27 (33.3%)	8 (38.1%)	19 (31.7%)	
1	41 (50.6%)	9 (42.9%)	32 (53.3%)	
2	9 (11.1%)	3 (14.3%)	6 (10.0%)	
3	4 (4.94%)	1 (4.76%)	3 (5.00%)	
4	1 (100%)	1 (100%)	0 (0%)	
Spirometry				
Pre‐BD				
FEV1/FVC (*n* = 154)	68.2 (14.5)	71.1 (14.0)	67.5 (14.6)	0.194
FEV_1_ (*n* = 154)	67.8 (20.6)	74.9 (21.9)	66.0 (19.9)	0.042
FVC (*n* = 154)	76.1 (21.5)	81.8 (20.0)	74.6 (21.8)	0.078
Post‐BD				
FVC (*n* = 189)	80.2 (22.9)	82.4 (26.6)	79.7 (22.0)	0.557
FEV_1_ (*n* = 189)	72.1 (21.7)	75.9 (22.6)	71.1 (21.4)	0.242
FEV_1_/FVC (*n* = 189)	68.3 (15.3)	69.8 (14.9)	67.9 (15.4)	0.496
Gold stages post‐BD, *n* (%)				0.277
GOLD 1 (*n* = 189)	39 (20.6%)	9 (23.7%)	30 (19.9%)	
GOLD 2 (*n* = 189)	125 (66.1%)	26 (68.4%)	99 (65.6%)	
GOLD 3 (*n* = 189)	23 (12.2%)	2 (5.26%)	21 (13.9%)	
GOLD 4 (*n* = 189)	2 (1.06%)	1 (2.63%)	1 (0.66%)	
Active treatment for COPD, *n* (%)				
SABA	96 (20.3%)	20 (19.0%)	76 (20.6%)	0.833
SAMA	189 (40%)	42 (40.0%)	147 (39.8%)	1
LABA	40 (8.44%)	8 (7.62%)	32 (8.67%)	0.886
LAMA	2 (0.42%)	0 (0%)	2 (0.54%)	1
LABA+LAMA	54 (11.4%)	10 (9.52%)	44 (11.9%)	0.611
ICS	32 (6.75%)	9 (8.57%)	23 (6.23%)	0.534
LABA+ICS	113 (23.8%)	26 (25%)	87 (23.6%)	0.903
LAMA+ICS	11 (2.32%)	4 (3.8%)	7 (2%)	0.271
LAMA+LABA+ICS	27 (5.6%)	5 (5%)	22 (6%)	
METHYLXANTHINE	2 (0.42%)	0 (0%)	2 (0.5%)	1
Inhaler adherence, *n* (%)				0.157
Acceptable adherence	46 (32.9%)	6 (19.4%)	40 (36.7%)	
Good adherence	89 (63.6%)	24 (77.4%)	65 (59.6%)	
Comorbidities, *n* (%)				
Anxiety	86 (18.1%)	33 (31.4%)	53 (14.4%)	< 0.001**
Arthritis	58 (12.2%)	14 (13.3%)	44 (11.9%)	0.826
Osteoarthritis	184 (38.8%)	48 (45.7%)	136 (36.9%)	0.126
Asthma	37 (7.81%)	16 (15.2%)	21 (5.69%)	0.003*
Stroke	45 (9.49%)	8 (7.62%)	37 (10.0%)	0.58
Ischemic heart disease	61 (12.9%)	10 (9.52%)	51 (13.8%)	0.32
Cardiomyopathy	13 (2.74%)	1 (0.95%)	12 (3.25%)	0.315
Depression	114 (24.1%)	46 (43.8%)	68 (18.4%)	< 0.001**
Diabetes	119 (25.1%)	16 (15.2%)	103 (27.9%)	0.012*
Arrhythmias	61 (12.9%)	11 (10.5%)	50 (13.6%)	0.506
Hypertension	260 (54.9%)	48 (45.7%)	212 (57.5%)	0.043*
Heart failure	58 (12.2%)	12 (11.4%)	46 (12.5%)	0.906
Chronic kidney disease	68 (14.3%)	12 (11.4%)	56 (15.2%)	0.419
Obesity	204 (43.0%)	44 (41.9%)	160 (43.4%)	0.878
Osteoporosis	31 (6.54%)	25 (23.8%)	6 (1.63%)	< 0.001**
Cardiac conduction disorder	56 (11.8%)	10 (9.52%)	46 (12.5%)	0.514
Thyroid disorder	58 (12.2%)	31 (29.5%)	27 (7.32%)	< 0.001**
Alcohol‐related disorder	98 (20.7%)	9 (8.57%)	89 (24.1%)	0.001**
Valvopathy	35 (7.38%)	13 (12.4%)	22 (5.96%)	0.045*
No chronic conditions	16 (3.38%)	3 (2.86%)	13 (3.52%)	1
GMA number of chronic diseases	9.56 (4.40)	10.4 (3.79)	9.33 (4.52)	0.016*
Healthcare resource utilisation				
Primary care visits				
General Practitioner	3.03 (3.15)	3.28 (3.30)	2.96 (3.10)	0.411
Nursing	2.96 (4.51)	2.73 (6.28)	3.02 (3.89)	0.673
Visits to other levels of care				
Emergency services	1.01 (1.83)	1.10 (2.42)	0.99 (1.62)	0.711
Emergency discharge	1.34 (1.67)	1.38 (1.72)	1.33 (1.65)	0.811
Urgent primary care centre (CUAP)	1.19 (2.22)	1.12 (2.00)	1.21 (2.28)	0.744
Hospital admission	1.23 (1.79)	1.44 (2.32)	1.16 (1.60)	0.357
GMA level, *n* (%)				0.188
1	19 (4.03%)	3 (2.86%)	16 (4.36%)	
2	44 (9.32%)	8 (7.62%)	36 (9.81%)	
3	235 (49.8%)	52 (49.5%)	183 (49.9%)	
4	174 (36.9%)	42 (40.0%)	132 (36.0%)	
Annual visits	20.7 (17.1)	22.3 (20.3)	20.3 (16.1)	0.361

*Note:* Variables marked with ** show highly significant differences between groups, while those marked with * indicate statistically significant differences. Variables without asterisks were not statistically significant.

With this limitation in mind, Table 1 shows that men had significantly higher height, weight than women (both *p* < 0.001), whereas BMI were only marginally higher in men and did not reach statistical significance (*p* = 0.053). (Table 1). A total of 52.3% of women and 45% of men were current smokers, although cumulative tobacco exposure (pack‐years) was higher in men. The level of smoking cessation intervention within PAPPS was relatively similar between genders, with behavioural reinforcement and intensive intervention being the most frequent, but fewer than 2% of patients received pharmacological treatment for smoking cessation (Table 1). Men reported higher alcohol consumption than women. The different clinical variables recorded were very similar in both groups. Dyspnea grade (mMRC) was documented in 20% of women and 16% of men, with no statistically significant difference between them. Most patients (both men and women) were classified as grade 0 or 1, indicating no or only mild dyspnea on exercise. However, it is worth noting the low rate of recording of this variable.

The EHR contained spirometric values for only 154 individuals pre‐bronchodilator (32%) and 189 individuals post‐bronchodilator (40%). This indicates a very high percentage of individuals ‘labelled as COPD’ without spirometric confirmation (Vila et al. 2025). On average, available spirometric values showed a mild obstructive ventilatory impairment in women and a moderate impairment in men, although with considerable interindividual variability. In fact, most patients presented a GOLD stage 1 (mild) – (20.6%) or GOLD 2 (moderate) – (66.1%), and only a 12.2% (mainly men) presented with GOLD 3 (severe) or GOLD 4 (very severe) – 1.1%. No significant bronchodilator reversibility was observed.

The most frequently prescribed inhaled treatments were short‐acting muscarinic antagonists (SAMA) and inhaled corticosteroid–based combinations (LABA+ICS), whereas LAMA monotherapy was infrequent. The use of oral methylxanthines was anecdotal (Table 1). Adherence to inhaled treatment was recorded in only a small proportion of the sample (30 women [28.5%] and 105 men [28.5%]), but it was adequate in most women (77.4%) and only in about half of the men (59.6%).

The most prevalent comorbidities were arterial hypertension (45.7% in women and 57.5% in men), obesity (41.9% and 43.4%), and osteoarthritis (45.7% and 36.9%). Other conditions, such as anxiety, depression, osteoporosis and thyroid disorders, were more frequent in women.

### 3.2 Stratification by GMA Complexity Group

**FIGURE 1:**
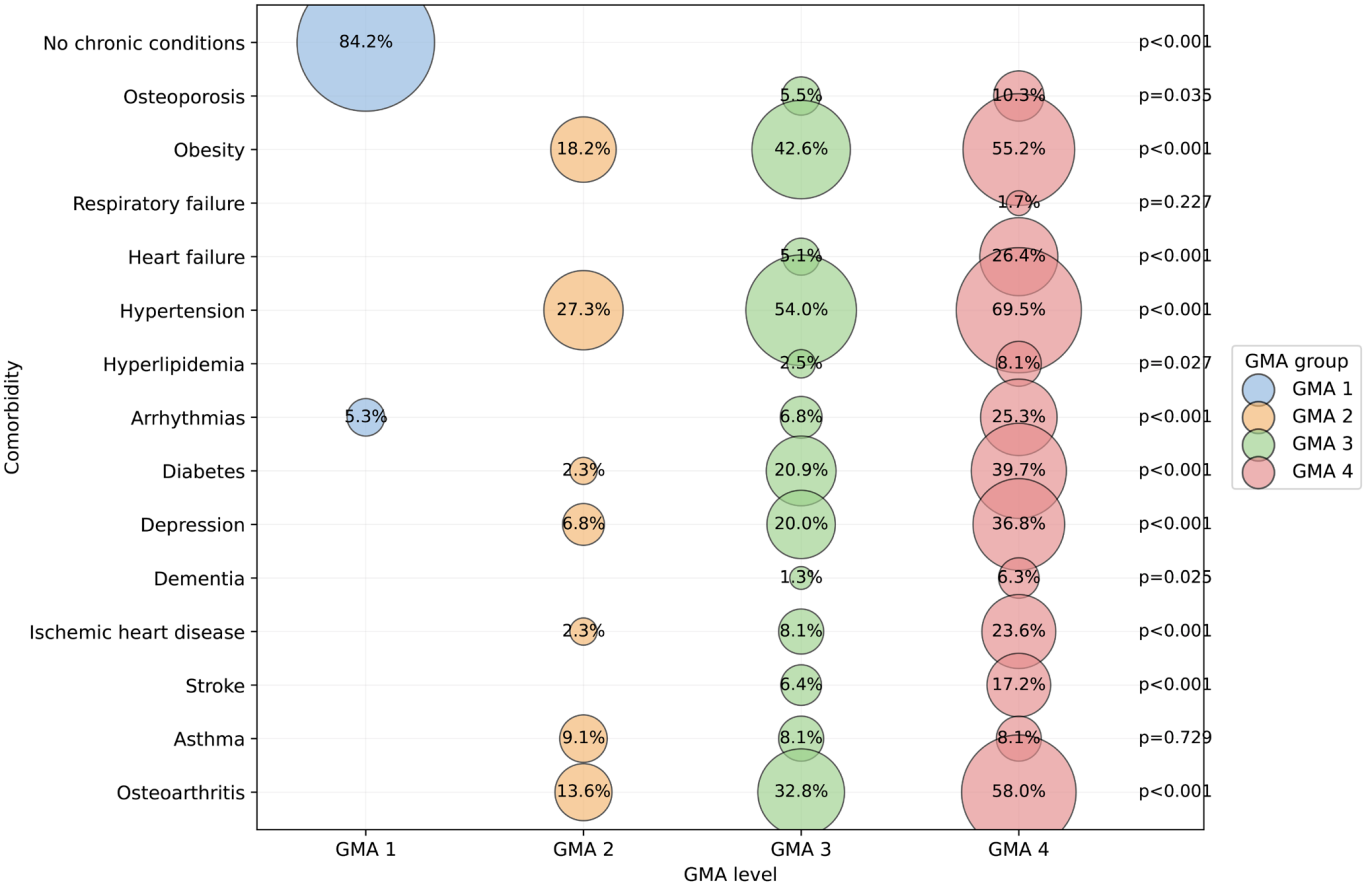
Representation of the most prevalent comorbidities (with a prevalence > 5%), in patients labelled with COPD in each GMA group. Circle size is proportional to prevalence. Proportions across GMA groups were compared using the χ^2^ test or Fisher's exact test when appropriate; displayed *p*‐values correspond to these tests. [Colour figure can be viewed at wileyonlinelibrary.com]

The Adjusted Morbidity Groups (GMA) index provides a weighted summary of patients' chronic disease morbidity, acute diagnosis codes and complexity (Monterde et al. 2016). As expected, Figure 1 illustrates the progressive increase in comorbidity burden, from GMA 1, characterised by the absence of chronic disease (84.2%), to GMA 4, where osteoarthritis (58.0%), hypertension (69.5%) and obesity (55.2%) predominate (*p* < 0.001). In GMA 2, hypertension (27.3%) and obesity (18.2%) stand out, while in GMA 3, hypertension (54.0%) and obesity (42.6%) are the most prevalent (*p* < 0.001). Cardiovascular conditions (hypertension and obesity), osteoarthritis and metabolic disorders commonly appear across most GMA groups (except GMA 1), with a higher prevalence than other comorbidities, as shown in Figure 1.

Table 2 compares the clinical characteristics of GMA groups 1–4 (from least to most complex). No significant differences were observed in sex distribution, with men predominating in all groups. Mean age increased progressively from GMA 2 to GMA 4. Primary care utilisation varied across GMA categories (Table 2). GP and nursing visits showed a non‐monotonic pattern at lower levels, with slightly higher values in GMA1 than in GMA2, and increased from GMA2 to GMA4 (*p* < 0.001). In contrast, visits to other levels of care did not differ significantly across GMA groups. Spirometric parameters did not show statistically significant differences between GMA groups.

**TABLE 2:** Comparison of clinical and healthcare characteristics according to GMA level.

	GMA 1	GMA 2	GMA 3	GMA 4	*p*
	*N* = 19	*N* = 44	*N* = 235	*N* = 174	
Sex					0.805
Woman	3 (15.8%)	8 (18.2%)	52 (22.1%)	42 (24.1%)	
Man	16 (84.2%)	36 (81.8%)	183 (77.9%)	132 (75.9%)	
Age	65.5 (16.7)	61.5 (9.29)	68.4 (10.9)	74.2 (9.48)	< 0.001**
Spirometry (pre‐post BD)					
Pre‐BD					
FEV1/FVC (*n* = 154)	70.2 (10.3)	78.9 (19.1)	77.6 (19.0)	74.1 (25.9)	0.688
FEV1 (*n* = 154)	60.5 (13.9)	67.6 (24.6)	69.3 (18.8)	66.5 (23.3)	0.709
FVC (*n* = 154)	69.6 (16.4)	63.7 (17.6)	68.9 (13.2)	67.8 (15.8)	0.793
Post‐BD					
FVC (*n* = 189)	80.2 (19.8)	80.0 (18.9)	81.5 (26.1)	78.1 (17.8)	0.831
FEV1 (*n* = 189)	69.8 (8.77)	67.2 (23.9)	73.0 (21.9)	71.8 (21.8)	0.823
FEV1/FVC (*n* = 189)	69.4 (17.5)	63.2 (17.8)	68.3 (15.5)	69.3 (14.4)	0.627
Current smoker	8 (61.5%)	23 (74.2%)	102 (50.2%)	56 (35.7%)	< 0.001**
Pack‐years	. (.)	46.6 (9.21)	28.4 (17.5)	29.2 (38.9)	0.467
Primary care visits					
General Practitioner	2.17 (1.92)	1.37 (2.05)	2.55 (2.40)	4.17 (3.91)	< 0.001**
Nursing	1.39 (1.24)	1.11 (1.25)	1.86 (2.24)	4.99 (6.35)	< 0.001**
Visits to other levels of care					
Emergency services	1.00 (1.41)	0.84 (1.61)	0.84 (1.58)	1.20 (2.09)	0.455
Emergency discharge	1.11 (0.93)	1.00 (1.86)	1.19 (1.52)	1.55 (1.79)	0.238
Urgent care centre (CUAP)	1.22 (1.30)	0.95 (1.27)	1.05 (2.36)	1.36 (2.23)	0.663
Hospital admission	0.78 (1.09)	0.95 (1.96)	1.03 (1.77)	1.48 (1.81)	0.153
GMA complexity	0.27 (0.74)	5.94 (1.18)	12.2 (2.39)	22.7 (5.20)	< 0.001**
GMA number of chronic diseases	0.12 (0.34)	3.93 (1.54)	8.17 (2.15)	13.6 (3.18)	< 0.001**
Annual visits	15.1 (17.4)	9.02 (10.6)	16.1 (11.3)	30.0 (20.1)	< 0.001**

*Note:* Variables marked with ** show highly significant differences between groups, while those marked with * indicate statistically significant differences. Variables without asterisks were not statistically significant.

The proportion of current smokers was higher in GMA 1 and 2 (61.5% and 74.2%, respectively), decreasing progressively in GMA 3 and 4 (50.2% and 35.7%). The number of primary care visits increased in parallel with complexity, especially nursing consultations. Likewise, the total number of annual visits was significantly higher in GMA 4. No relevant differences were found in the use of emergency services, urgent care centres or hospital admissions. As expected, both the GMA complexity index and the number of chronic conditions clearly increased from GMA 1 to GMA 4. In our setting, GMA stratification is used to describe patients' clinical complexity and patterns of care. In this study, GMA was applied for descriptive stratification only, and no protocolised interventions were imposed according to GMA categories.

### 3.3 Healthcare Resource Utilisation and Comorbidities

**FIGURE 2:**
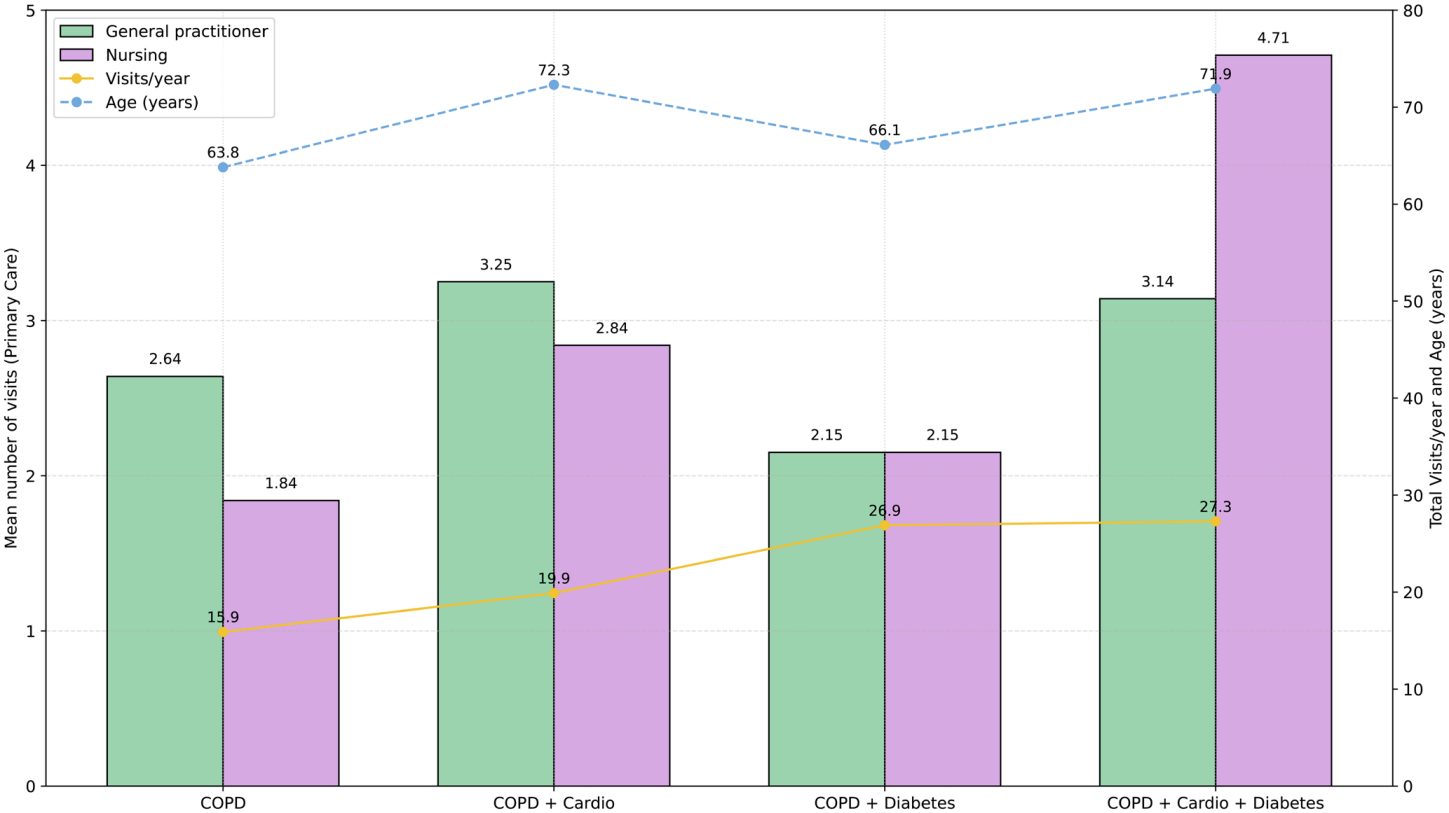
Comparison of resource use and profile by group. Bars: Average number of primary care visits (family doctor in green, nurse in purple). Lines: Total visits/year (yellow) and average age (blue; right axis). Four profiles are compared: COPD, COPD + cardiovascular, COPD + diabetes and COPD + cardiovascular + diabetes. There were more nursing visits and more visits/year in the cardiometabolic profiles (*p* < 0.001), with no differences in the family doctor profiles (*p* = 0.272). [Colour figure can be viewed at wileyonlinelibrary.com]

Figure 2 compares healthcare resource utilisation according to comorbidities: COPD, COPD with cardiovascular disease, COPD with diabetes and COPD with both cardiovascular disease and diabetes. At variance with medical visits, nursing visits differed significantly between these groups (*p* < 0.001), with higher values in COPD patients with both cardiovascular disease and diabetes (4.71 ± 4.87) vs. COPD with cardiovascular disease only (2.84 ± 3.74; *p* = 0.004) vs. COPD alone (1.84 ± 5.27; *p* < 0.001), whereas this figure was similar in patients with COPD with diabetes or COPD alone.

Total annual visits (i.e., any primary care visit, including medical visits, chronic care follow‐up, PAPPS, complementary tests, etc.) were also higher in COPD with both cardiovascular disease and diabetes (27.3 ± 15.9; *p* < 0.001) and in COPD with diabetes (26.9 ± 16.5; *p* < 0.001) compared with COPD alone (15.9 ± 17.1; *p* < 0.001 and *p* = 0.063, respectively). No significant differences were observed in the use of other health care services, such as emergency discharges, urgent primary care centres (CUAP) or hospital admissions (*p* > 0.05).

## 4 Discussion

This descriptive database study provides three main observations of interest for nursing primary care follow‐up of patients with COPD: (1) the prevalence of patients labelled as COPD in the EHR is very low, and many of them do not have spirometric diagnostic confirmation; (2) there is substantial under‐recording in the EHR of key follow‐up variables (e.g., level of dyspnea, spirometric variables and inhaler use); and (3) patients with ‘COPD’ and cardiovascular disease and/or diabetes are followed up more closely in nursing (not in medical) consultations than those with COPD alone.

### 4.1 Previous Studies

Several previous studies have already reported significant gaps in the completeness of the EHR in PC (Schaubroeck et al. 2020) (Llauger et al. 2014), particularly in relation with the results (if ever measured) of spirometry, a key variable in the diagnosis and management of COPD (Boman et al. 2010; Van De Hei et al. 2020). Our results here confirm these previous observations and extend them to the specific domain ‐for example inhaler‐technique checks‐ of nursing consultations in PC. To our knowledge, this is the first study specifically assessing the recording and follow‐up of nursing care plans within primary care, thereby highlighting a previously unexplored dimension of EHR completeness.

Previous research has highlighted the central role of primary care nursing in the longitudinal management of COPD and other chronic diseases, particularly in patient education, inhaler technique assessment, smoking cessation support and monitoring of symptoms and treatment adherence (Harries and White 2021). However, several studies have reported that this contribution is often under‐recognised or insufficiently documented in routine clinical records (Hjort et al. 2025; Verde‐remeseiro et al. 2015). Our findings are consistent with these observations, suggesting that nursing activity related to COPD may be substantially underestimated when relying solely on structured EHR data, especially amongst patients with lower recorded complexity.

### 4.2 Interpretation of Results

As previously described, the spirometry‐defined prevalence of COPD in the general population is ~10% in adults aged ≥ 40 years; in our cohort, the prevalence of EHR‐labelled COPD was substantially lower (GOLD 2025b). Several factors can account for such huge discrepancy. First, we included individuals older than 15 years in whom the prevalence of COPD is much lower; however, the mean age of the identified individuals was about 70 years of age (Table 1). Second, many of these ‘labelled COPD’ individuals did not have spirometric confirmation; yet it is likely that, if they have had it, the prevalence of true COPD may have been even lower. Actually, this contrasts very significantly with a previous national general population study in the city of Manlleu (also in the same region of Osona studied here) that in 2000 reported a prevalence of spirometrically confirmed airflow obstruction of 18% in individuals over 40 years of age vs. about 9% in the rest of Spain (Sobradillo Peña et al. 2000). So, if anything, we should have expected a higher prevalence of ‘COPD’ in our study here. Finally, we also found that many data fields were incomplete in the EHR—for example; smoking cessation stage (*n* = 59/474; 12.4%); dyspnea scale—mMRC (*n* = 82; 17.3%); respiratory rate (*n* = 86; 18.1%); inhaler adherence (*n* = 135; 28.5%); spirometry pre‐BD—FEV_1_/FVC, FEV_1_, FVC (*n* = 154; 32.5%); and GOLD stage (*n* = 189; 39.9%); oxygen saturation (*n* = 211; 44.6%) ‐, so we believe that the observed prevalence of labelled COPD here (1.7%) is biassed and does not reflect the true situation in the general population of our region. However, it clearly illustrates the deficiencies existing in the diagnosis and recording of relevant data in the EHR in PC. In fact, part of the information may have been entered as unstructured free text and therefore could not automatically be retrievable. Perhaps the future use of artificial intelligence tools (e.g., large language models) may help to improve retrieval of such information. This situation is not unique to our region. In a previous study in a larger PC population in Catalonia we observed the same pattern: only 54% of patients labelled as COPD had spirometry recorded in the EHR (Vila et al. 2025). Of note, spirometry confirmed the presence of airflow obstruction in only 29.5% of them, where the rest (24%) were misdiagnosed (i.e., no airflow obstruction is present). Recently, the new concept of ‘pre‐COPD’ has been proposed to identify individuals with symptoms and risk factors without airflow obstruction but having other structural or functional abnormalities, some of whom may progress to COPD (Han et al. 2021). In any case, this is highly relevant clinically because underdiagnosis implies no treatment, and misdiagnosis implies wrong treatment.

On the other hand, we observed patients labelled as COPD with other comorbidities (cardiovascular and/or diabetes) were followed up much more closely (both in medical and nursing consultations) than those without these (recorded) concomitant diseases (Figure 2). This may be directly due to the higher clinical complexity of the former, who really need such closer follow‐up, and/or to a certain lack of relevance for those patients having (recorded in the EHR) only a COPD diagnosis. The latter possibility is intriguing since it is well established that cardiovascular and metabolic comorbidities occur very often in patients with COPD (Fabbri et al. 2023; Celli et al. 2025). We postulate, therefore that again, this likely reflects under‐recording of relevant clinical information. Supporting this interpretation is the absence of significant differences in emergency visits, urgent care centres, and hospital admissions.

Finally, two other observations in our study may be worth discussing. First, almost half of these COPD labelled patients were still current smokers (Table 1), but smoking cessation interventions were rare. And second, the under‐recording of the level of dyspnea and adherence to medication limits the ability to adjust pharmacologic and non‐pharmacologic treatment and highlights an organisational need: standardised COPD templates in the EHR, interoperability with spirometers and regular data quality audits.

From a clinical and organisational perspective, these findings underscore the importance of improving the visibility and standardisation of nursing activities within the EHR (Bertocchi et al. 2025), as previously highlighted in studies on structured nursing documentation in COPD care (Hjort et al. 2025). Standardised COPD care templates, better integration of spirometric data, and structured recording of key variables such as dyspnea, smoking status and treatment adherence could facilitate more consistent follow‐up and support quality improvement initiatives. Importantly, our results should be interpreted descriptively and do not imply that care intensity is determined by GMA category, but rather that recorded complexity is associated with differences in documented follow‐up.

### 4.3 Strengths and Limitations

To our knowledge, this is the first study to investigate the clinical profile, comorbidity burden, follow‐up and healthcare utilisation in patients labelled in the EHR as COPD in PC nursing consultations. Amongst potential limitations, we acknowledge the retrospective design; potential under‐recording and documentation heterogeneity in the EHR; and that part of the information may have been entered as unstructured free text and therefore could not be automatically retrieved. Likewise, the small size of some subgroups (e.g., COPD with diabetes) reduces statistical power, and the absence of certain clinical variables (such as annual exacerbations or other exposures) as well as the lack of analysis of non‐respiratory medications (e.g., cardiovascular drugs), restrict causal inferences. In addition, spirometry was available in only a subset of patients (approximately 32%–40%), which introduces potential selection bias; therefore, the observed GOLD stage distribution should be interpreted only amongst patients with available spirometric data and should not be extrapolated to all patients recorded as having COPD in the EHR.

Finally, previous hospitalisations due to COPD exacerbations were not captured in the available data, although such events may represent an important determinant of disease management in primary care and the involvement of nursing consultations. Moreover, the descriptive and exploratory nature of the analyses, together with the absence of data imputation, adjustment for potential confounders, and correction for multiple comparisons, limit the interpretation of the findings to descriptive associations rather than causal relationships.

## 5 Conclusions

This study shows that the prevalence of patients labelled as COPD in the EHR in PC is very low, that many of them do not have spirometric diagnostic confirmation and that there is substantial under‐recording in the EHR of key follow‐up variables (e.g., level of dyspnea, spirometric variables and inhaler use). Accordingly, nursing consultations in PC offer a valuable (and so far not up to speed) opportunity to confirm (or exclude) the presence of COPD by spirometry, optimise treatment, address comorbidities and improve the recording and monitoring of clinical management of these patients. In this context, implementing standardised EHR templates and/or protocols, ensuring interoperability and integration with spirometers, and conducting regular evaluations led by nursing staff (prioritising high GMA groups) could improve quality and equity amongst COPD patients.

## Author Contributions

**Marc Vila:** conceptualisation (lead), original draft (lead), formal analysis (lead), review and editing (equal); **Meritxell Mondejar:** review and editing (equal); **Sergio Cazorla‐Calderón:** review and editing (equal); **Àngels Ballarin:** review and editing (equal); **Sandra Casas‐Recasens:** formal analysis and software (lead); **Rosa Faner:** supervision (lead), funding acquisition (lead), review and editing (equal); **Alvar Agustí:** conceptualisation (senior), supervision (lead), funding acquisition (lead), review and editing (equal).

## Funding

Project PM21/00090, Instituto de Salud Carlos III (NextGeneration EU), ‘Mechanism for Recovery and Resilience’ (MRR)/PRTR.

## Ethics Statement

The study was conducted in accordance with the ethical standards of the 1975 Declaration of Helsinki. The study protocol was reviewed and approved in April 2024 by the *Research Ethics Committee on Medicines of the Institute for Research and Innovation in Life and Health Sciences in Central Catalonia* (CEIm IRIS‐CC) (approval code: 24/027).

## Consent

Informed consent was waived, as only anonymised data were used, ensuring patient confidentiality and preventing the identification of individual participants.

## Conflicts of Interest

The authors declare no conflicts of interest.

## Supporting information


**Data S1:** STROBE Checklist.

## Data Availability

The data that support the findings of this study are available on request from the corresponding author. The data are not publicly available due to privacy or ethical restrictions.
