# Supplementary material for: COPD Management in Primary Care: Underutilisation of Nursing Consultations
Source: J Clin Nurs. 2026 Feb 12;35(6):2744–54. doi: 10.1111/jocn.70241 (PMC13156530; doi:10.1111/jocn.70241)
Supplement: Supplementary file 1 — Data S1: STROBE Checklist. [file JOCN-35-2744-s001.pdf]

STROBE Statement—Checklist of items that should be included in reports of ***cross-sectional studies***

	Item No	Recommendation	Page No
**Title and abstract**	1	(*a*) Indicate the study's design with a commonly used term in the title or the abstract	1,2
		(*b*) Provide in the abstract an informative and balanced summary of what was done and what was found	2
**Introduction**			
Background/rationale	2	Explain the scientific background and rationale for the investigation being reported	Line 40
Objectives	3	State specific objectives, including any prespecified hypotheses	Line 60
**Methods**			
Study design	4	Present key elements of study design early in the paper	Line 70
Setting	5	Describe the setting, locations, and relevant dates, including periods of recruitment, exposure, follow-up, and data collection	Line 88
Participants	6	(*a*) Give the eligibility criteria, and the sources and methods of selection of participants	Line 79
Variables	7	Clearly define all outcomes, exposures, predictors, potential confounders, and effect modifiers. Give diagnostic criteria, if applicable	Line 92
Data sources/ measurement	8*	For each variable of interest, give sources of data and details of methods of assessment (measurement). Describe comparability of assessment methods if there is more than one group	-
Bias	9	Describe any efforts to address potential sources of bias	-
Study size	10	Explain how the study size was arrived at	Line 79 and line 130
Quantitative variables	11	Explain how quantitative variables were handled in the analyses. If applicable, describe which groupings were chosen and why	Line 92
Statistical methods	12	(*a*) Describe all statistical methods, including those used to control for confounding	Line 114
		(*b*) Describe any methods used to examine subgroups and interactions	Line 114
		(*c*) Explain how missing data were addressed	Line 114
		(*d*) If applicable, describe analytical methods taking account of sampling strategy	Line 114
		(*e*) Describe any sensitivity analyses	Line 114
**Results**			
Participants	13*	(a) Report numbers of individuals at each stage of study—eg numbers potentially eligible, examined for eligibility, confirmed eligible, included in the study, completing follow-up, and analysed	Line 130
		(b) Give reasons for non-participation at each stage	-
		(c) Consider use of a flow diagram	-
Descriptive data	14*	(a) Give characteristics of study participants (eg demographic, clinical, social) and information on exposures and potential confounders	Line 132
		(b) Indicate number of participants with missing data for each variable of interest	-

Outcome data	15*	Report numbers of outcome events or summary measures	Line 128
Main results	16	(*a*) Give unadjusted estimates and, if applicable, confounder-adjusted estimates and their precision (eg, 95% confidence interval). Make clear which confounders were adjusted for and why they were included	Line 128
		(*b*) Report category boundaries when continuous variables were categorized	Line 128
		(*c*) If relevant, consider translating estimates of relative risk into absolute risk for a meaningful time period	Line 128
Other analyses	17	Report other analyses done—eg analyses of subgroups and interactions, and sensitivity analyses	Line 128
**Discussion**			
Key results	18	Summarise key results with reference to study objectives	Line 218
Limitations	19	Discuss limitations of the study, taking into account sources of potential bias or imprecision. Discuss both direction and magnitude of any potential bias	Line 218
Interpretation	20	Give a cautious overall interpretation of results considering objectives, limitations, multiplicity of analyses, results from similar studies, and other relevant evidence	Line 218
Generalisability	21	Discuss the generalisability (external validity) of the study results	Line 218
**Other information**			
Funding	22	Give the source of funding and the role of the funders for the present study and, if applicable, for the original study on which the present article is based	Page 1.

*Give information separately for exposed and unexposed groups.

Note: An Explanation and Elaboration article discusses each checklist item and gives methodological background and published examples of transparent reporting. The STROBE checklist is best used in conjunction with this article (freely available on the Web sites of PLoS Medicine at http://www.plosmedicine.org/, Annals of Internal Medicine at http://www.annals.org/, and Epidemiology at http://www.epidem.com/). Information on the STROBE Initiative is available at www.strobe-statement.org.
